# The Association Between Obesity and Long-Term Cognitive Performance in Middle-Aged and Older Adults

**DOI:** 10.1093/geroni/igaa057.1222

**Published:** 2020-12-16

**Authors:** Andrew Fiscella, Ross Andel

**Affiliations:** University of South Florida, Tampa, Florida, United States

## Abstract

Obesity is a growing epidemic in the United States and has been associated with negative health outcomes such as cardiovascular disease and diabetes. However, an obesity paradox has emerged which suggests that the effects of obesity may vary by age, with older adults potentially seeing a protective effect of obesity. This study examined the effects of overweight and obese status on cognitive performance at baseline and follow-up. It was hypothesized that obese middle-aged adults would perform worse than normal weight peers, but that reverse would be observed in older adults. Data from 701 participants in the Midlife in the United States study were included. Body mass index (BMI) and waist circumference were employed as measures of obesity. Z-scores for executive function, memory, and global cognition were used to quantify cognitive performance. While obese participants tended to perform worse on average than normal weight individuals there were no significant differences in performance between obese and normal weight participants in global cognition (p=.134), executive function (p=.164), or episodic memory (p=.708). Additionally, age did not moderate this relationship. However, there was a significant effect of education on all three domains. When stratified by education, participants with some college or higher, had a significant time*obesity*age interaction (F[3,328]=3.016, p<.05). For the oldest-old participants, executive function scores were higher for obese participants at follow-up compared to normal weight participants, but not at baseline. These findings suggest that level of education may serve as a form of cognitive reserve which compensates for deficits due to obesity.

